# A Model of Academic, Personality, and Emotion-Related Predictors of University Academic Performance

**DOI:** 10.3389/fpsyg.2019.02435

**Published:** 2019-11-05

**Authors:** Maria-Jose Sanchez-Ruiz, Jamil El Khoury

**Affiliations:** Department of Social Sciences, School of Arts and Sciences, Lebanese American University, Byblos, Lebanon

**Keywords:** academic performance, trait emotional intelligence, personality, academic motivation, major satisfaction, procrastination, higher education

## Abstract

This paper investigates the relationships between personality (i.e., trait Emotional Intelligence – trait EI – and the Big Five) and academic performance (AP). Academic motivation, procrastination, and major satisfaction were also studied. The sample consisted of 201 Lebanese undergraduates. The model represented a good fit. There was a negative direct effect of procrastination on AP and positive direct effects of major satisfaction and absorption on AP. Trait EI showed a negative direct effect on procrastination and a positive direct effect on major satisfaction, which, in turn, significantly predicted AP. Also, conscientiousness indirectly predicted AP, via procrastination, major satisfaction, and absorption. Findings point at individual differences contributing to AP and can be helpful to students, educators, and counselors in higher education.

## Introduction

The factors contributing to one individual’s achievement over another’s in educational settings is an issue of extensive debate and continues to draw vast investigative interests (e.g., Tavani and Losh, 2003). However, evidence is mixed on the extent to which cognitive ability predicts academic achievement versus personality factors (Bratko et al., 2006; Boerchi et al., 2018). A number of studies have concluded that cognitive ability is a strong predictor of academic performance (AP; Kuncel and Hezlett, 2010), while others have indicated incremental validity of personality traits over cognitive ability (Sanchez-Ruiz et al., 2013).

With the large body of research currently available on the role of personality traits in influencing AP (e.g., Petrides et al., 2004; Laidra et al., 2007; Perera and DiGiacomo, 2013; Boerchi et al., 2018), the conception that cognitive and task-specific abilities are the only, if not the most, significant factors in predicting achievement has become questionable.

Although research so far has led to mixed findings (Mavroveli and Sanchez-Ruiz, 2011), the study by Sanchez-Ruiz et al. (2016) has shown that trait Emotional Intelligence (*trait EI* or *emotional self-efficacy*) has implications on AP, with effects mainly relevant to groups with lower cognitive ability (see Petrides et al., 2018, for a review). Perera and DiGiacomo (2013) reported the validity of trait EI in predicting AP, and Parker et al. (2016) added more evidence on the link between the two variables. Trait EI is a constellation of emotional perceptions and inherent qualities at the low-lying levels of personality structures, and it is measured by Likert-scale questionnaires (Petrides et al., 2007).

Studies have focused their attention mainly on the Big Five personality traits in relation to AP. Research by Petrides et al. (2007) has shown that trait EI is distinct from the Big Five and comprehensively measures the facets of personality related to emotion. Therefore, with the growing body of evidence revealing a possible significant interaction between emotion-related personality constructs, specifically the construct of trait EI, and AP, measured by GPA (Grade Point Average), this paper will include trait EI alongside the Big Five personality traits in examining their effects on AP.

Contrary to trait EI, which has been shown to moderate the effect of stress (Mikolajczak et al., 2006), procrastination is viewed as a maladaptive coping strategy against academic stress (Alexander and Onwuegbuzie, 2007), with detrimental effects on AP (De Paola and Scoppa, 2015). A study conducted by Deniz et al. (2009) reported that adaptability and coping with stress, which are subscales of EI, and overall EI were negatively associated with procrastination. These findings suggest that trait EI can facilitate self-management in demanding educational environments, which protects against procrastination and might consequently promote AP.

Very few studies have investigated the relationship between trait EI and major satisfaction. One such study was conducted by Urquijo and Extremera (2017), who found there was a positive association between EI and academic satisfaction, though not exactly satisfaction with the chosen field of study. Other personality and emotion-related constructs such as academic engagement have also been found to contribute to AP (Boerchi et al., 2018; Vizoso et al., 2018) and psychological well-being (Ayyash-Abdo and Sanchez-Ruiz, 2012). In the study by Vizoso et al. (2018), all three dimensions of academic engagement (i.e., vigor, dedication and absorption) were positively related to AP. AP, then, is best viewed as a product of the interaction between cognitive as well as personality processes (Chamorro-Premuzic and Furnham, 2005).

### The Present Study

The uniqueness of the present study lies in that, to our knowledge, it is the first study that brings the studied variables together in the same research design. Moreover, research on academic outcomes in Lebanon is sparse and outdated, and has focused specifically on differential analysis of academic achievement between American and Asian students (Baran, 2008), and grade retention in relation to academic history (El-Hassan, 1998). To our knowledge, there are no studies exploring the relationship between emotion-related variables and AP in the Lebanese context. The present study aims to contribute in this direction.

The central aim of this study is to achieve a better understanding of how individual differences in personality and emotion-related traits (with special emphasis on trait EI) play a role in AP. In addition, we are interested in investigating the contribution of other personality variables, namely, procrastination, academic engagement, satisfaction with university major, and personality (Big Five) to AP. In this paper, we explore the associations among these variables and present a model of direct and indirect effects on AP using structural equation modeling. The novelty of the present study is twofold. First, it presents a model to explain AP in relation to non-cognitive variables such as trait EI and academic motivation in the context of higher education. Second, it contributes to the limited research on emotion-related variables and AP using a Lebanese sample.

## Materials and Methods

### Participants

Participants consisted of 201 (131 females) Lebanese university students. The mean age of the participants was 19.76 (*SD* = 1.85) years old. The students were enrolled in majors related to arts and humanities (26%), technical and/or natural sciences (35%), business (27%), and social sciences (12%), and were either freshmen (10%), sophomore (35%), junior (26%), or senior (27%).

### Measures

#### Academic Performance

Academic performance was measured using each student’s self-reported current university GPA. The GPA is measured on a scale ranging from 0 to 4, with 4 being the highest grade.

#### Trait Emotional Intelligence

The *Trait Emotional Intelligence Questionnaire – Short Form* (TEIQue-SF; Petrides, 2009). The TEIQue-SF includes statements such as “I often pause and think about my feelings.” Participants responded to all survey items using a seven-point Likert scale (from 1 = “Disagree strongly” to 7 = “Agree strongly”). On this sample, the internal reliabilities for Well-being, Self-control, Emotionality, Sociability, and global trait EI were 0.82, 0.50, 0.67, 0.52, and 0.86, respectively.

#### Big Five Personality Traits

The *International Personality Item Pool* (IPIP; Goldberg, 1999). Items include “I see myself as someone who has an assertive personality.” Participants responded to all survey items using a five-point Likert scale (from 1 = “Disagree strongly” to 5 = “Agree strongly”). The internal reliabilities for this sample were 0.80 for extraversion, 0.71 for agreeableness, 0.78 for conscientiousness, 0.83 for emotionality, and 0.74 for openness to experience.

#### Academic Engagement

The English version of the *Utrecht Work Engagement Student Scale* (UWES-SS; Schaufeli et al., 2002). The Scale of Academic Engagement is a 17-item questionnaire. Participants responded to all survey items using a seven-point Likert scale (from 0 = “Never” to 6 = “Always”). The items represent three underlying dimensions: Vigor (e.g., “when I get up in the morning, I feel like going to class”), Dedication (e.g., “I am enthusiastic about my studies”), and Absorption (e.g., “when I am studying, I forget everything around me”). In this sample, the internal reliabilities obtained for this scale were 0.89 (total score), 0.72 (Vigor), 0.64 (Dedication), and 0.80 (Absorption).

#### Procrastination

The *General Behavioral Procrastination Scale* for student populations (GPS; Lay, 1986). The GPS consists of 20 items, including “I generally delay before starting on work I have to do.” Participants responded to all survey items using a five-point Likert scale (from 1 = “Extremely uncharacteristic” to 5 = “Extremely characteristic”). The alpha coefficient for this scale on the present sample was 0.88.

#### Satisfaction With University Major

Satisfaction with university major was measured through a one-item question, which asked “How satisfied are you with the major you are in?” and was responded to on a seven-point Likert scale (from 1 = “Extremely dissatisfied” to 7 = “Extremely satisfied”).

### Procedure

This study has been approved by the Institutional Review Board (IRB) of ethics at the Lebanese American University. Participants completed the battery of questionnaires during class time. A small percentage of the participants were given the questionnaires after class, which they returned to the researchers after completion. Testing sessions lasted 50 min approximately and were monitored by the researchers. Data were analyzed using SPSS version 25, and its AMOS module.

## Results

### Bivariate Correlations

Intercorrelations among the study variables are presented in Table 1. GPA positively correlated with conscientiousness (*r* = 0.23, *p* = 0.004), absorption (*r* = 0.27, *p* = 0.001), and major satisfaction (*r* = 0.29, *p* < 0.001), and negatively with procrastination (*r* = –0.26, *p* = 0.001). Global trait EI was positively associated with conscientiousness (*r* = 0.29, *p* < 0.001) and major satisfaction (*r* = 0.23, *p* = 0.004), and negatively with procrastination (*r* = –0.28, *p* < 0.001). Conscientiousness positively correlated with absorption (*r* = 0.32, *p* < 0.001) and major satisfaction (*r* = 0.22, *p* = 0.005), and negatively with procrastination (*r* = –0.58, *p* < 0.001).

**TABLE 1:** Bivariate correlations among study variables (*N* = 160).

	**1**	**2**	**3**	**4**	**5**	**6**	**7**	**8**	**9**	**10**	**11**
(1) GPA											
(2) Extraversion	–0.07										
(3) Agreeableness	0.08	0.17^∗^									
(4) Conscientiousness	0.23^∗∗^	0.06	0.21^∗∗^								
(5) Emotional stability	–0.05	–0.05	0.00	0.01							
(6) Openness to experience	0.19^∗^	0.16^∗^	0.07	0.22^∗∗^	–0.01						
(7) Global trait EI	–0.01	0.39^∗∗∗^	0.14^∗^	0.29^∗∗∗^	0.57^∗∗∗^	0.33^∗∗∗^					
(8) Procrastination	–0.26^∗∗^	0.04^∗^	–0.07	–0.58^∗∗∗^	–0.06	–0.02^∗∗^	–0.28^∗∗∗^				
(9) Vigor	0.20^∗∗^	0.08	0.07^∗∗^	0.32^∗∗∗^	0.01	0.32^∗∗∗^	0.19^∗∗^	–0.34^∗∗∗^			
(10) Dedication	0.19^∗^	0.06	0.05	0.17^∗^	–0.04	0.26^∗∗^	0.09^∗∗^	–0.21^∗∗^	0.68^∗∗∗^		
(11) Absorption	0.27^∗∗^	0.06^∗^	0.07	0.32^∗∗∗^	–0.03	0.29^∗∗∗^	0.04^∗∗^	–0.29^∗∗∗^	0.81^∗∗∗^	0.69^∗∗∗^	
(12) Major satisfaction	0.29^∗∗∗^	0.04	0.04	0.22^∗∗^	0.11	0.07^∗^	0.23^∗∗^	–0.24^∗∗^	0.32^∗∗∗^	0.30^∗∗∗^	0.26^∗∗^

*^∗∗∗^*p* < 0.001, ^∗∗^*p* < 0.01, ^∗^*p* < 0.05.*

### Path Analysis

#### Model Fit

**FIGURE 1:**
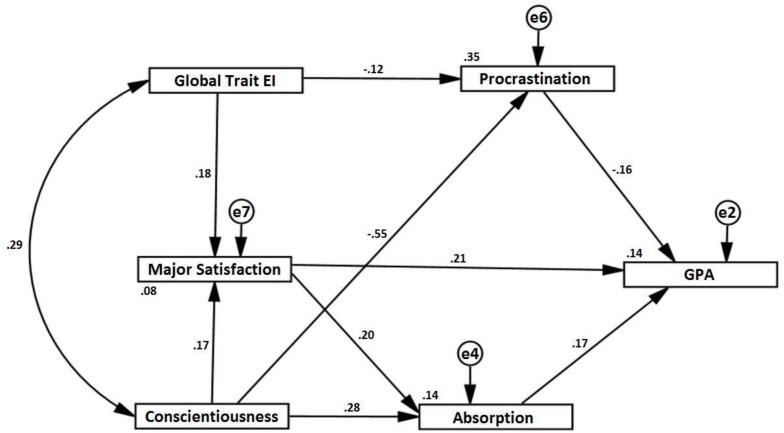
Hypothesized model with the respective path coefficients.

The model tested included indirect effects between global trait EI and GPA, via procrastination, and major satisfaction. Conscientiousness was hypothesized to have indirect effects on GPA, with procrastination, major satisfaction, and absorption as mediators. Major satisfaction and absorption were expected to have a positive direct effect on GPA. Conscientiousness and global trait EI were allowed to covary, as they have shown to relate in previous research. Figure 1 illustrates the model with the respective path coefficients. The following indices were used to assess the model fit (Byrne, 2010): the overall chi-square statistics and relative/normed chi-square (CMIN/df), with values below 2 and *p* > 0.05 indicating good fit; the Comparative Fit Index (CFI), with values above 0.90 indicating good fit; the Standardized Root Mean Square Residual (SRMR), with values below 0.08 indicative of good fit; and the Root Mean Square Error of Approximation (RMSEA), with values between 0 and 0.05 indicative of good fit, between 0.05 and 0.08 indicative of acceptable fit, and above 0.1 indicative of poor fit; and the respective closeness of fit (PCLOSE), with a *p* > 0.05. Based on the above indices, the model hypothesized revealed a good fit with an acceptable RMSEA, χ^2^(5, 160) = 9.048, *p* > 0.05, CMIN/df = 1.810, CFI = 0.971, SRMR = 0.0448, and RMSEA = 0.071, PCLOSE > 0.05.

#### Direct and Indirect Effects

Procrastination, absorption, and major satisfaction had significant direct effects on GPA, with β = –0.164, *p* < 0.05, β = 0.170, *p* < 0.05, and β = 0.211, *p* < 0.01, respectively. There was a significant direct effect from global trait EI to major satisfaction (β = 0.181, *p* < 0.05), and a marginally significant direct effect from global trait EI to procrastination (β = –0.117, *p* < 0.10). Conscientiousness had a significant effect on procrastination (β = –0.548, *p* < 0.001), major satisfaction (β = 0.167, *p* < 0.05), and absorption (β = 0.277, *p* < 0.001). Major satisfaction had a significant direct effect on absorption (β = 0.195, *p* < 0.05). Conscientiousness and global trait EI were correlated positively (*r* = 0.290, *p* < 0.001). The *R*^2^ indicates that the model accounts for 14% of the variance in GPA.

## Discussion

The hypothesized model revealed the indirect effects of global trait EI on AP via procrastination and major satisfaction, and the indirect effect of conscientiousness on AP via procrastination, major satisfaction, and absorption. The model explained 14% in the total variance of AP with a good fit, which is considerably high when compared to previous similar models exploring indirect effects on AP via personality traits, ranging from 6 to 14% (McIlveen et al., 2013).

### Direct Effects

Our results showed that global trait EI negatively predicted procrastination. The delay feature of procrastination lies within the students’ self-control (Ackerman and Gross, 2005), and a method of coping with stress (Alexander and Onwuegbuzie, 2007), with a negative association with EI (Deniz et al., 2009). Furthermore, procrastination has been conceptualized as a form of self-regulatory failure, with a consistent negative relation with self-efficacy (Steel, 2007). Students with high global trait EI have “superior emotion information processing skills, regulation, and coping skills” and may be more successful in coping with the demands of school and peer context (Mavroveli and Sanchez-Ruiz, 2011). Therefore, high trait EI is directly incompatible with the self-regulatory deficiency associated with higher procrastination.

In addition, global trait EI had a direct effect on major satisfaction. This adds to the scarce evidence on this link, which might be driven by certain facets of trait EI such as optimism (Logue et al., 2007), which, in turn, positively correlates with AP (Martirosyan et al., 2014). High trait EI students have greater self-knowledge, which affords higher capabilities in making wiser vocational and occupational decisions that are compatible with their personality; therefore, conducive to personal and academic satisfaction (see Sanchez-Ruiz et al., 2010 for a brief discussion on this idea).

Conscientiousness negatively predicted procrastination in the model, in line with previous studies (e.g., Steel, 2007). This can be explained by the fact that conscientiousness implies self-discipline, acting dutifully, and preference for planning. In support of this claim, a study revealed punctuality as one of the behavioral indicators of conscientiousness (Jackson et al., 2010).

Furthermore, the direct association between conscientiousness and major satisfaction was positive (e.g., Kirwan et al., 2014). Because conscientious students strive for achievement and are more responsible (Clark and Schroth, 2010), they are more likely to choose majors that are compatible with their interests and in which they excel at (Denissen et al., 2007).

The model showed a positive direct effect between conscientiousness and absorption. Previous studies conducted on work engagement found similar results between the two variables (Kim et al., 2009), which suggests that such a relation could also be relevant in academic engagement. In addition, the association between conscientiousness and effort strategies (Corker et al., 2012), and task focus (Saklofske et al., 2012), may reflect the high academic absorption conscientious students preserve.

The direct effects on AP were all significant. The results showed that procrastination has an aversive effect on AP, as with previous studies (Kennedy and Tuckman, 2013). Similar to our findings, previous studies revealed a positive relation between AP and major satisfaction (Martirosyan et al., 2014), and academic engagement (Casuso-Holgado et al., 2013).

### Indirect Effects of Trait EI and Conscientiousness

The indirect effects of global trait EI and conscientiousness on AP, via major procrastination, were both significant. This might indicate that the two personality traits contribute as protective factors against maladaptive coping, such as procrastination, in an academic context. Besides procrastination, absorption also mediated the effect between conscientiousness and AP, which highlights the importance of such a trait regarding concentration, attention, and engagement while studying.

Global trait EI and conscientiousness had an indirect effect on AP, via major satisfaction. The indirect effects of lower-order personality traits, such as trait EI, and higher-order personality traits, such as conscientiousness, on AP serve as personality predispositions to aim for higher levels of compatibility between the student’s personality and academic major choice and in turn higher performance.

### Implications, Limitations, and Future Directions

Our results shed light on the importance of personality traits such as trait EI and conscientiousness for success in higher education. Our findings can inform education professionals who, knowing the individual predictors of AP for their students, can use this information to develop strategies that reinforce the underlying behaviors associated with such traits. For example, instructors might want to promote conscientious behaviors, which in turn might have an impact on the student’s engagement and satisfaction, and subsequently on higher levels of achievement. Likewise, career counseling practitioners can use this information to develop implementation programs to foster adaptive emotion-related approaches, which have shown to be effective (e.g., Slaski and Cartwright, 2003) to orient students toward affective and behavioral coping when experiencing academic stress (Mavroveli and Sanchez-Ruiz, 2011) and increase engagement and satisfaction in university studies. This can be done under the umbrella of Emotional Education, which is a current interest within professional and research arenas (e.g., Sanchez-Ruiz et al., 2010). Also, our results can assist the development of interventions and guidance services for students at risk of academic difficulties (McKenzie and Schweitzer, 2001).

This study has several limitations. First, this is a cross-sectional study conducted on a convenience sample. Second, all variables are assessed using self-report measures, which allows for the possibility of mono-method bias. Longitudinal, mixed-method designs on larger, and more diverse samples would increase validity. In addition, it is advisable to use official transcripts from educational institution whenever available, as a more reliable estimation of the GPA than self-reported data. Future research can also benefit from incorporating objective measures (e.g., cognitive ability) and potential moderators of the relationships under investigation, such as major of study. Lastly, upcoming studies could explore the relationship between trait EI and AP (and its correlates) at the factor level because trait EI has shown to relate differently to a variety of constructs depending on whether the focus is on the sociability, emotionality, self-control, or well-being factor (e.g., Sanchez-Ruiz et al., 2011.

## Conclusion

This study provides preliminary data that can serve to develop a thorough and parsimonious predictive model of AP of practical utility in educational assessment and counseling in higher education. Finally, this study has contributed to the scarce research on emotion-related personality dispositions, and specifically trait EI, in the educational context in the Lebanese population.

## Data Availability Statement

The datasets generated for this study are available on request to the corresponding author.

## Ethics Statement

The studies involving human participants were reviewed and approved by the Institutional Review Board at the Lebanese American University. The participants provided their written informed consent to participate in this study.

## Author Contributions

M-JS-R designed the study, coordinated the data collection, cleaning, and analysis, reviewed the theoretical and empirical background, and drafted the section “Discussion.” JE critically reviewed the relevant literature and contributed to the sections “Materials and Methods” and “Results.” Both authors wrote and reviewed the manuscript.

## Conflict of Interest

The authors declare that the research was conducted in the absence of any commercial or financial relationships that could be construed as a potential conflict of interest.
